# Interactive Dyadic Physical Activity and Spatial Proximity Patterns in 2-Year-Olds and Their Parents

**DOI:** 10.3390/children5120167

**Published:** 2018-12-11

**Authors:** Aston K. McCullough, Helena Duch, Carol Ewing Garber

**Affiliations:** 1 Department of Biobehavioral Sciences, Columbia University Teachers College, New York, NY 10027, USA; ceg2140@tc.columbia.edu; 2 Department of Population and Family Health, Mailman School of Public Health, Columbia University, New York, NY 10032, USA; helena.duch@oakfnd.ch

**Keywords:** children, adult, physical activity, family interaction

## Abstract

This study aimed to characterize daily physical activity (PA) behaviors in 2-year-old girls and boys and their parents, with and without an objective measure of dyadic spatial proximity. Urban-dwelling parent–toddler dyads (*N* = 110) wore accelerometers for 7 days, and parents completed a sociodemographic questionnaire. Accelerometers were initialized to collect PA and Bluetooth-based proximity data. After applying wear-time algorithms, *n* = 65 dyads were further analyzed using a dyadic analysis statistical methodology. Toddler–parent sedentary and light PA time were respectively interdependent, conditional on child sex and child-parent proximity, but moderate–vigorous physical activity (MVPA) time was not. Toddlers were significantly more active on weekdays and weekends than their parents, and no differences were found in daily PA volumes between girls and boys. In dyads with proximity data (*n* = 34), analyses of joint (i.e., proximal and mutual) PA time showed that girls participated in significantly more joint PA with their mothers than boys. Children who engaged in ≥60 min of MVPA/day participated in ~2 h of joint PA/day, on average, while children with <60 min of MVPA/day engaged in ~30 min less joint-PA time with their mothers. Boys and girls who participated in higher daily MVPA volumes engaged in joint PA with their mothers across greater relative distances, as compared to less active boys who engaged in joint PA at closer relative distances to their mothers. Toddlers who engaged in ≥60 min of daily MVPA participated in joint PA with their mothers at greater relative distances and for longer durations than less active children. Further research on the dyadic activity–proximity relationship is needed across early childhood development.

## 1. Introduction

Early childhood (2–5 years old) is an essential period during physical activity (PA) behavior development [1], and PA is an important correlate of health in the early years [2]. Research shows that parents play an important role in early childhood PA development and the years beyond [1,3], with a growing number of studies on child and parent PA confirming positive associations between child-parent habitual daily PA volumes throughout childhood and well into adulthood [4,5]. While a large body of evidence appears to support an interdependent child-parent PA relationship, research also shows that these dyadic PA interdependencies may not be ubiquitous [6,7]. As such, further research is needed to better understand the interdependent child-parent PA relationship and contributing factors.

Studies of dyadic PA in young children and their parents have reported differential associations between child and parent PA after stratifying analyses by child and parent sex [6,8]. However, little is known about what contributes to these differences in the dyadic PA relationship between boys, girls, and their parents. A recent review of methods for measuring child and parent co-participation in PA has suggested the use of accelerometry in tandem with an objective measure of child-parent proximity to provide more robust descriptions of child-parent PA [9]. Such a multi-sensor approach captures dyadic PA in terms of both dyadic activity intensities and spatial dynamics [9], which in their combination may help to reveal interpersonal factors associated with the child-parent PA relationship at a key developmental stage.

Few studies, however, have measured child and parent PA and dyadic proximity in early childhood using wearable technology [7,9]. In a sample of 1–5-year-olds and their mothers, no differences in joint child-parent PA volumes were found between mothers and their children with respect to child sex [7]. By contrast, a report of objectively measured child-parent PA and proximity in older children (8–14-year-olds) showed that child sex was associated with differences in joint child-parent PA volumes [10]. Given the potential influences of interpersonal proximity, child sex, and parent sex on the child-parent PA relationship, the need for further research on dyadic PA and proximity during early childhood is evident. This need is further underscored by the overall paucity of objective dyadic PA-proximity reports that are currently available to inform the developing definition of child and parent co-participation in PA [9].

Therefore, the aims of this descriptive study were to measure dyadic physical activity and interpersonal spatial proximity in 2 year-old boys and girls and their parents using wearable technology in order to characterize: (1) habitual daily child-parent PA interdependence, with and without a measure of dyadic proximity, (2) hour-to-hour interactive child-parent PA interdependence over a 3 day period, and (3) joint physical activity behaviors in child-parent dyads.

## 2. Materials and Methods

### 2.1. Site and Sample

Study participants were recruited from an Early Head Start (EHS) in a major urban center, with a catchment area that serves under-resourced families across multiple city districts. EHS is a nationally funded program that aims to bolster the physical, cognitive, and socio-emotional development of children under 3 years and their parents. The EHS reported that 57% of its families share apartments with ≥1 family and 95% of families speak Spanish in the home. Families attend the EHS center for ~3.5 h once per week, and receive semi-monthly home-visits from one of their regular classroom teachers. Parents of 24–35-month-old children attending the EHS were invited to participate in this study. All children who were 24–35 months-old without exclusion criteria were eligible for the study and were invited to participate. Exclusion criteria included children with extreme developmental delays, significant sensory or behavioral concerns, or health conditions that might restrict PA. The absence of exclusion criteria was confirmed by parental or teacher report at the time of recruitment. Parents provided informed consent according to the policies and procedures of the Columbia University Medical Center and Columbia University Teachers College Institutional Review Boards that approved the study protocol.

### 2.2. Measures

#### 2.2.1. Physical Activity

Child-parent dyads were asked to wear triaxial ActiGraph accelerometers (Pensacola, FL, USA; Models: wGT3X+ or wGT3X-BT) at the hip (anterior superior iliac spine) for 7 contiguous days [11,12], except while bathing or during sleep. Accelerometers were initialized to collect triaxial data at 30 Hz for 8 days [13]. ActiGraph data were exported from ActiLife software in 15 s epochs for children and 60 s epochs for parents. Data reduction procedures were conducted in MATLAB R2017a [14]. To yield accelerometer data with sufficient reliability (*r* ≥ 0.70), the non-wear-time criteria applied to children’s accelerometer data were: 0 cpm × 20 min, <6 h·day^−1^, <3 days observed [12]. For adults, non-wear-time criteria were: 0 cpm × 90 min, 120 s spike tolerance (bracketed by 30 min spike tolerance windows), <6 h·day^−1^, <4 days observed [15,16], where cpm refers to the counts observed per minute. Accelerometer data were analyzed for *N* = 110 parent-child pairs, and after applying data reduction algorithms, *n* = 65 dyads met wear-time criteria and were included in further analyses. Activity intensity thresholds for sedentary behavior (SED), light PA (LPA), and moderate–vigorous PA (MVPA) were applied to accelerometer counts for children (SED (≤25); LPA (>25 and <420); MVPA (≥420)) and adults (SED (≤99); LPA (<99 and <1952); MVPA (≥1952)) using established cut points [17,18]. The proportion of parents meeting current recommendations for MVPA of ≥150 min/week were calculated [19]. The proportion of children meeting current recommendations for total physical activity (TPA) and MVPA of ≥180 min/day and ≥60 min/day, respectively, were also calculated [20,21]. TPA was defined as any activity above the respective SED thresholds for children and parents.

#### 2.2.2. Proximity

Proximity tagging data that are available in newer accelerometers can be used as an objective measure of dyadic proximity [9]. ActiGraph wGT3X-BT accelerometers were additionally initialized to collect proximity data in 60 s epochs, with devices respectively assigned as “receiver” and “beacon” for child-parent dyads [7]. Accelerometer-derived proximity data can be used to predict metered distances between devices (≤20 m), as well as to yield proportions of time that devices are proximal (≤50 m) [22]. Proximity data were conserved for periods during which dyads had mutually valid accelerometer wear-time data. Proportions of time when receivers and beacons were within range were calculated. Metered distances between dyadic counterparts were also predicted from accelerometer-derived Bluetooth signals [22] and were then transformed into z-scores to derive a standardized measure of the relative distance (i.e., nearer (<0) or farther (>0)) between child and parent during activity. Of the dyads meeting wear-time criteria, *n* = 34 dyads had proximity data and were included in further proximity analyses. All dyads with proximity data were also mother-child dyads.

Considering the current recommendation for a consensus on the definition of *co-participation in PA* [9], we focused on a single element of the current understanding of the term—mutual and proximal engagement in activity. We use the term *joint* to describe such bouts of activity wherein child and parent activity intensities are synchronous and dyadic counterparts are oriented proximally in space.

#### 2.2.3. Additional Measures

To compute descriptive statistics of sample characteristics and to also adjust models of child-parent activity and proximity for potentially influential covariates, parents were asked to complete a brief sociodemographic questionnaire. The questionnaire included items on children (age and sex) and parents (age, sex, country of origin, household income, education, and family size). 

### 2.3. Statistical Analyses

A dyadic analysis methodology was employed for this study because it is expressly suited for studies wherein data have been purposively sampled in dyads [23]. Moreover, the application of a dyadic analysis methodology to child-parent PA data affords researchers an opportunity to analyze interactive child-parent PA patterns using over-time dyadic models, which can provide high-level detail on child-parent PA interdependencies at various temporal resolutions.

Data were analyzed in MATLAB and MPLUS 7 [24]. Descriptive statistics are reported as Mean (Standard Deviation; SD), Median (Interquartile Range; IQR), or Frequencies (%) for child-parent sociodemographic characteristics (*M*(SD), Frequencies), activity behavior (M(SD), Frequencies), and dyadic proximity data (Mean(SD), Median(IQR), Frequencies). To test for interdependence between child and parent covariates of interest, Pearson’s correlations (*r*) and their 95% Confidence Intervals (95%CI) were calculated for child and parent proportions of time spent SED, in LPA, and in MVPA [23]. Pearson’s correlations were repeated after stratifying by child sex. Correlations between the proportion of time that children and parents spent in each respective activity threshold (SED, LPA, and MVPA) while proximal were also examined after stratifying by child sex.

Partial correlations were tested between the proportions of time that children and parents spent in TPA, after controlling for children’s age and the mean daily proportion of time that dyads were proximal. The following linear mixed effects (LME) models were run after controlling for the random effect of dyad—Model 1: Weekday and weekend TPA ~ day (i.e., weekday and weekend), role (i.e., child and parent), day × role, child sex, and child age. Model 2: included proximity and a proximity × sex interaction as additional terms to those in Model 1. Continuous covariates were mean centered to facilitate interpretation of interaction effects. A residual maximum likelihood method was used in LME models with a sample size of *n* ≤ 50, otherwise a maximum likelihood estimator was used [25]. Respective models were systematically assessed for the assumptions of (1) within dyad interdependence [23] and (2) mutually independent residuals with distribution *N*~(0,*σ*^2^)—both of which were found to be tenable across models [25]. The random intercept only model showed that >5% of the variance in TPA was explained by the effect of dyad. All beta coefficients (*β*) are presented with their standard error (*SE*).

Interactive hour-to-hour child and parent TPA behaviors were examined in an actor-partner interdependence model (APIM) [23]. The APIM estimates cross-partner influences within dyads and the stability of a given signal within subjects over time. Dyadic hour-to-hour proportions of time spent in TPA were randomly extracted from 3 monitored days on which children and parents mutually had valid TPA data. A total of *n* = 63 dyads had sufficient mutual wear-time data and were included in the APIM analysis. Model 3: For a given hour, child TPA time (CHILD_t_) was regressed on lagged child (CHILD_t−1_) and lagged parent (PARENT_t−1_) TPA time, and parent TPA time (PARENT_t_) was regressed on lagged child and lagged parent TPA time. To explore the influence of hourly dyadic proximity on hourly TPA time, an additional model was fit for dyads with proximity data. Again, here the model averaged over TPA hour-to-hour, and thus does not distinguish between periods of mutual engagement versus non-mutual engagement in PA during each hour. Additionally, the proportion of time spent in proximity hour-to-hour only represents the amount of time that dyadic counterparts spent together each hour and does not distinguish between periods of activity or inactivity. Model 4: The lagged proportion of time that children’s and parents’ devices were within range (PROX_t−1_) was added to the model, such that CHILD_t−1_ and PARENT_t−1_ TPA were regressed on PROX_t−1_, and PARENT_t_ and CHILD_t_ were regressed on PARENT_t−1_ and CHILD_t−1._ Continuous covariates were mean centered; the grand mean of CHILD_t−1_ and PARENT_t−1_ was respectively subtracted from each predictor according to APIM specifications in both models [23]. Both models were specified as nested models, as above, wherein observations could cluster within dyads and within subjects [23]. Model fit was assessed for both Model 3 ($\chi_{1}^{2}=0.71,\;p>0.05$; CFI/TLI ≥ 1.0; RMSEA < 0.01) and Model 4 ($\chi_{3}^{2}=3.64,\;p>0.05$; CFI/TLI ≥ 0.99; RMSEA = 0.01) and both were found to have good fit.

To explore differences in joint TPA with respect to child sex and MVPA, two separate two-way ANOVA models were run with *post hoc* multi-comparisons using Scheffe’s procedure. Model 5: Tested for differences in the proportion of time that children and parents engaged in joint TPA time, conditioning on the following dichotomous independent variables—child sex (female; male) and mean daily MVPA volume (<60 min/day; ≥60 min/day). Model 6: Tested for differences in the mean relative distance (z-scores) at which child and parent engaged in joint TPA, conditioning on child sex and mean daily MVPA volume. Main effects and interaction effects were assessed in each respective model. The assumptions of normality and homogeneity of variances were assessed and found to be tenable. Effect sizes are presented as partial eta squared. 

Tests for differences in MVPA and TPA time between dyads with and without proximity data showed that there were no significant differences for children nor parents. All models were estimated with an *a priori* significance level of *α* = 0.05.

## 3. Results

Summaries of sociodemographic variables and descriptive statistics for wear-time, activity, and proximity data are presented in Table 1.

Of those who reported their ethnicity (*n* = 43), >97% (42) identified as Latino/Hispanic and ~2% (1) identified as not Latino/Hispanic. Overall, children spent an average 52(8)% of their time SED, 38(5)% in LPA, and 10(4)% in MVPA, while parents spent 56(9)% SED, 40(8)% in LPA, and 4(2)% of their time in MVPA. Dyadic counterparts were within proximity 73(18)% of mutually monitored wear time, on average, with a relative mean distance z-score of −0.01(0.21). Table 2 shows activity and proximity characteristics for girls, boys, and their mothers while their respective dyadic counterpart was engaged in activity of any given intensity. With respect to overall wear time, dyads engaged in joint SED a mean 19(7)% of the time with a mean relative distance z-score of −0.01(0.22), in joint MVPA <0.01(0.005)% of the time with a relative distance z-score of −0.001(0.46), and in joint TPA 15(5)% of the time with a relative distance z-score of 0.14(0.54).

### 3.1. Dyadic Activity–Proximity Interdependencies

#### 3.1.1. Without Proximity

Pearson’s correlations showed that the mean daily proportions of time that children and parents spent SED each day were not significantly correlated (*r* = 0.20, 95%CI: −0.05 to 0.43, *p* > 0.05), nor were the proportions of time spent in MVPA (*r* = −0.07, 95%CI: −0.31 to 0.18, *p* > 0.05); however, mean daily proportions of time spent in LPA were significantly correlated (*r* = 0.27, 95%CI: 0.03 to 0.48, *p* = 0.03). After stratifying by child sex, no relationship (*p* > 0.05) was found between the overall proportions of time that girls and their parents spent SED (*r* = 0.03, 95%CI: −0.31 to 0.37), in LPA (*r* = 0.09, 95%CI: −0.26 to 0.42), or MVPA (*r* = −0.06, 95%CI: −0.39 to 0.28) each day. The proportions of time that boys and their parents spent SED (*r* = 0.45, 95%CI: 0.11 to 0.69, *p =* 0.01) and in LPA (*r* = 0.44, 95%CI: 0.10 to 0.69, *p* = 0.03) were interdependent, while boy-parent MVPA was not (*r* < 0.01, 95% CI: −0.35 to 0.36, *p* > 0.05).

#### 3.1.2. With Proximity

Pearson’s correlations showed that the mean proportions of time that children and their mothers spent SED were interdependent when dyadic counterparts were proximal (*r* = 0.78, 95%CI: 0.60 to 0.89, *p* < 0.001). The mean proportions of time spent in LPA were also interdependent when child and mother were proximal (*r* = 0.75, 95%CI: 0.55 to 0.87, *p* < 0.001); however, the mean proportions of time spent in MVPA were not interdependent when child and mother were proximal (*r* = −0.13, 95%CI: −0.45 to 0.22, *p* > 0.05).

When girls and their mothers were proximal, the mean proportions of time spent SED were interdependent (*r* = 0.90, 95%CI: 0.72 to 0.97, *p* < 0.001), mean LPA volumes were interdependent (*r* = 0.87, 95%CI: 0.65 to 0.96, *p* < 0.001), and the mean proportions of time spent in MVPA were not interdependent (*r* = 0.17, 95%CI: −0.38 to 0.63, *p >* 0.05). Among boys and their mothers, the mean proportions of time spent SED were interdependent when proximal (*r* = 0.72, 95%CI: 0.40 to 0.89, *p* = 0.001), the mean proportions of time spent in LPA were interdependent (*r* = 0.47, 95%CI: 0.02 to 0.76, *p* = 0.04), and the mean proportions of time spent in MVPA were not interdependent (*r* = −0.26, 95%CI: −0.64 to 0.22, *p >* 0.05).

Partial correlations showed that the proportions of time that child and mother spent in TPA while proximal (*ρ* = 0.73, 95%CI: 0.42 to 0.86, *p* < 0.001) remained positively associated after controlling for age and the relative TPA distance z-scores for mothers and their children.

### 3.2. Overall Daily Child-Parent PA

#### 3.2.1. Without Proximity

Results from Model 1 showed that children spent 7(1)% (*p* < 0.001) more time in weekday and weekend TPA than parents (42(1)%), controlling for all other Model covariates. Dyads were also 2(1)%, more active on weekdays (*p* = 0.02) than weekends. Dyadic weekend and weekday TPA were not associated with child age nor sex (Adjusted *R*^2^ = 0.39).

#### 3.2.2. With Proximity

After adjusting for the proportion of time that child-mother dyads were in proximity and the proximity × child sex interaction, there was a significant interaction between proximity and sex (*β_prox_* × *β_sex_* = 0.22(0.07), *p* = 0.004). For a 1% increase in the proportion of time that girls and their mothers were within proximity, there was a 0.13% decrease in the mean proportion of time that girl-parent dyads spent in TPA (52(6)%). For boys and their mothers, a 1% increase in the proportion of time that dyadic counterparts spent in proximity was associated with a 0.23% decrease in the mean proportion of time that boy-mother dyads spent in TPA (56(7)%). Role was also a significant covariate in the model (*p* < 0.001), showing again that, on average, children spent additional time in TPA (8(2)%) above parents (41(2)%), controlling for all other model covariables. No other model covariates were significantly associated with dyadic weekday and weekend TPA (Adjusted *R*^2^ = 0.40).

### 3.3. Hour-to-Hour Interactive Child-Parent PA

#### 3.3.1. Without Proximity

Model 3 showed that on average children’s (*β*_11_ = 0.37(0.02), *p* < 0.001) and parents’ (*β*_22_ = 0.47(0.02), *p* < 0.001) own TPA behaviors during a given hour significantly predicted their own TPA behavior in the following hour after adjusting for all other model covariates. Parents’ TPA during a given hour was also positively associated with their children’s TPA over time (*β*_21_ = 0.09(0.02), *p* < 0.001); however, children’s lagged TPA was not significantly associated with parents’ hourly TPA (*p* > 0.05). Children’s and parents’ hourly TPA were also correlated from hour-to-hour (*φ* = 0.29(0.02), *p* < 0.001), and the variances were also correlated for child-parent hourly TPA (*p* < 0.001).

#### 3.3.2. With Proximity

**Figure 1:**
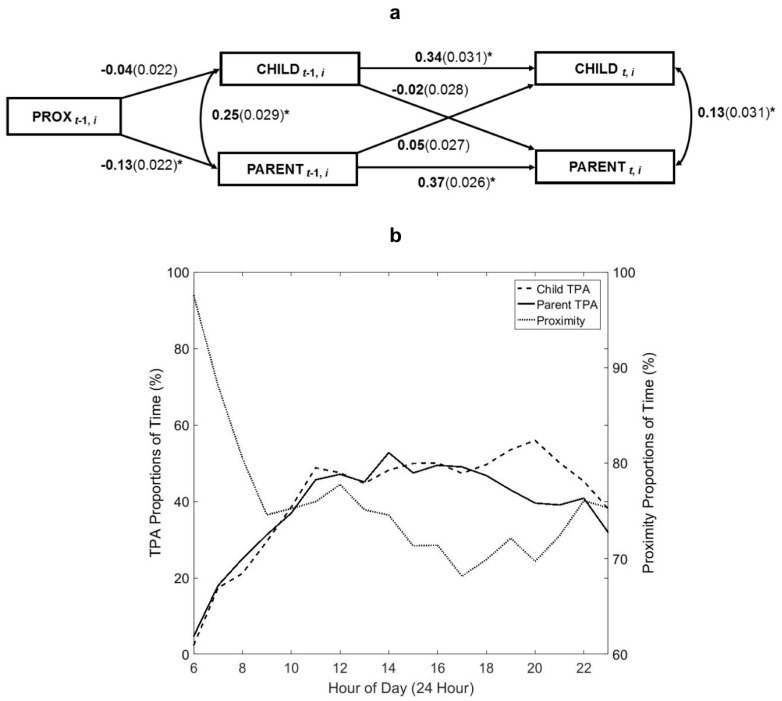
Associations between toddler-mother hourly physical activity time and dyadic proximity over 72 h among urban-dwelling families. Note: Figure 1**a** shows that children’s and mothers’ time spent in total physical activity (TPA) during a given hour significantly predicted (*p* < 0.01) their respective time spent in TPA during the following hour after controlling for dyadic proximity. The model also shows an inverse relationship between hour-to-hour dyadic proximity and maternal PA. Figure 1**b** illustrates hourly proximity-activity signals for child-parent dyads. Asterisk (*) indicates *p* < 0.01. Abbreviations: prox (proximity).

After adjusting for hourly dyadic proximity, dyadic hourly TPA remained correlated hour-to-hour (*φ* = 0.25, *p* < 0.001); however, mothers’ TPA no longer predicted children’s PA hour-to-hour (Figure 1a). Lagged hourly dyadic proximity was significantly inversely associated (*p* < 0.001) with lagged mothers’ TPA (*β* = −0.13) at a given hour, but not children’s (*p >* 0.05). Figure 1b shows dyadic mean hourly TPA and proximity signals throughout the day. The peak TPA time for parents was from 14:00 to 14:59, with parents spending (52.7(15.2)%) of their time in TPA on average and was from 20:00 to 20:59 for children (56.0(14.1)%). The mean proportion of time that dyads spent in proximity was lowest from 17:00 to 17:59 (68.2(21.0)%).

### 3.4. Joint Dyadic PA

#### 3.4.1. Daily Activity Time

**Figure 2:**
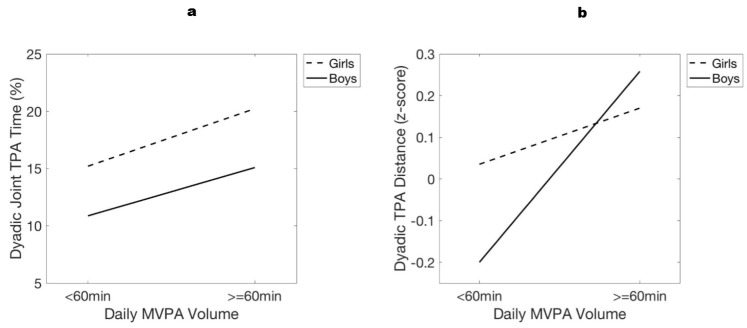
Differences in child-mother joint physical activity time (**left**) and relative distances (**right**). Note: Figure 2**a** shows that girls spent more time in joint PA with their mothers than boys, and children who engaged in ≥60 min MPVA/day participated in more joint PA than those with <60 min MVPA/day. Figure 2**b** shows that girls and boys who participated in ≥60 min MVPA/day engaged in joint PA with their mothers across wider relative distances, compared to boys with <60 min MVPA day who participated in joint PA at closer relative distances. Abbreviations: physical activity (PA), moderate–vigorous PA (MVPA), total PA (TPA), minutes (min).

The two-way ANOVA (Figure 2a) on the daily proportion of time children and mothers spent engaged in joint TPA showed a non-significant child sex × MVPA volume interaction (*F*_1,30_ = 0.07, *p* > 0.05, $\eta_{p}^{2}<0.01$), but significant main effects for child sex (*F*_1,30_ = 9.14, *p* < 0.01, $\eta_{p}^{2}=0.23$) and MVPA volume (*F*_1,30_ = 8.69, *p* < 0.01, $\eta_{p}^{2}=0.23$). *Post hoc* analyses showed that, on average, girls engaged in significantly more (*p* = 0.006) joint TPA time with mothers (18(1)%) than boys (13(1)%), and children who engaged ≥60 min of daily MVPA participated in significantly more (*p* = 0.005) joint TPA time with their mothers (18(1)%) than those with <60 min of daily MVPA (13(1)%).

#### 3.4.2. Dyadic Distance

Figure 2b shows results from the two-way ANOVA on the relative distance (z-scores) between child and mother while engaged in joint TPA. There was a significant child sex × MVPA volume interaction (*F*_1,30_ = 4.37, *p* < 0.05, $\eta_{p}^{2}=0.13$), a significant main effect for MVPA volume (*F*_1,30_ = 14.7, *p* < 0.001, $\eta_{p}^{2}=0.33$), and a non-significant main effect of child sex (*F*_1,30_ = 0.9, *p* > 0.05, $\eta_{p}^{2}=0.03$). *Post hoc* analyses showed that, on average, girls who participated in ≥60 min of daily MVPA engaged in joint TPA at significantly (*p* = 0.04) greater distances from their mothers (0.17(0.10)) than boys with <60 min of daily MVPA (−0.20(0.08)), as did boys with ≥60 min of daily MVPA (0.26(0.07)) with respect to boys with less MVPA (*p* = 0.001). Girls with <60 min of daily MVPA did not engage in joint TPA at significantly different distances (0.03(0.07)) than any other group (*p* > 0.05), and there were no significant differences in relative dyadic distances between boys and girls with ≥60 min of daily MVPA (*p* > 0.05).

## 4. Discussion

This study aimed to characterize the dyadic activity–proximity relationship in a sample of 2-year-old boys and girls and their parents. Using a dyadic analysis statistical methodology, results showed that child and parent mean daily SED and LPA, but not MVPA behaviors, were interdependent. These results, however, were conditional on child sex and child-parent proximity. Hour-to-hour dyadic PA behaviors were also interdependent after controlling for dyadic proximity, which was inversely associated with maternal hourly PA. Results also showed that children who engaged in higher daily MVPA volumes participated in greater amounts of joint (i.e., mutual and proximal) TPA time with their mothers than children with lower daily MVPA volumes. Moreover, with respect to joint TPA time, engaging in higher volumes of daily MVPA for children was associated with participating in dyadic PA across greater distances when compared to less active children. However, joint PA results were also conditional on child sex. Overall, the findings from our study suggest that interpersonal spatial dynamics between dyadic counterparts may help to explain some of the variability in early childhood PA, specifically with respect to boys’ and girls’ daily activity volumes and intensities.

At the dyadic level, child-mother daily PA was inversely associated with the proportion of time that counterparts were proximal, and child sex was found to moderate the inverse relationship between dyadic PA and proximity. However, further analyses of hour-to-hour activity–proximity data revealed that the inverse relationship between dyadic proximity and PA was only significantly associated with maternal hourly PA, and that it had no association with children’s hour-to-hour PA behaviors. It is important to note, however, that our model made no constraints on mutuality with respect to child and mother activity intensities at any given hour. As such, our data suggest that mothers were less active as they and their children spent more time in proximity, irrespective of their counterpart’s level of activity, which included proximal sedentary time. In another study, of objectively measured activity and proximity in young children and their mothers, joint TPA time was inversely associated with mothers’ PA at times when they were not proximal to their children [7]. Though direct comparisons between studies may be limited due to differences in proximity data processing approaches [7,22], both point toward inverse associations between dyadic proximity and maternal PA behaviors. Thus, further research is needed to better explain influential factors in the observed inverse relationship among mothers, as well as to confirm its presence across diverse dyadic cohorts. Additionally, child and parent hour-to-hour PA variances in our study remained correlated between counterparts after adjusting for within-subject hourly PA tracking, dyadic cross-partner interactions, and dyadic proximity. The remaining shared dyadic variance may point toward genetic, cultural, and environmental factors that were unexplained by the model [1,26]. As such, more research is needed to respectively characterize the relative contributions of learned, inherited, and environmental factors on the development of PA behavior in early childhood.

Targeted evaluation of the time periods during which children and mothers were engaged in joint PA revealed that toddlers’ daily MVPA volumes were associated with differences in child-mother joint PA time. Specifically, children in our study who engaged in higher daily volumes of moderate–vigorous PA (≥60 min/day) also spent an average 18% of their time (~2 h) in joint TPA with their mothers each day, as where children with lower daily MVPA volumes (<60 min/day) spent 13% of their time (~1.5 h) in joint PA with their mothers on average daily. These results are especially striking in light of the null relationship between child-mother MVPA time, and appear to suggest that children’s independent MVPA time is associated with joint child-mother total PA. Additional research is needed on familial co-participation in PA [9], especially toward considering joint PA recommendations in the early childhood years when PA modeling is considered an important feature of PA development [1,3]. Finally, given the average 30 min difference in joint PA between groups, these results invite research on the benefits of ~30 min joint PA activity interventions in young children and parents, particularly as they grow older and PA recommendations widely suggest participation in ≥60 min of daily MVPA [21].

Furthermore, our novel analysis of relative distance scores between dyadic counterparts while engaged in joint PA showed that child-mother spatial dynamics were associated with differences in toddlers’ daily MVPA volumes. Specifically, girls and boys who engaged in ≥60 min MVPA/day also participated in joint PA at greater distances from their mothers; this particular activity–proximity dynamic was especially important for boys. Among girls with lower daily MVPA volumes, however, there were no differences in the relative distances at which they participated joint TPA when compared to any other group. Notably, girls in our study were also found to participate in more joint PA with their mothers than boys. A prior report of dyadic PA and proximity in 8–14-year-olds and their parents also showed that girls spent more time in joint MVPA with their parents than boys [10], which similarly points toward sex-dependent differences in the dyadic activity relationship [6]. Despite differences between boys and girls with respect to child-mother joint PA time, no differences were found between the overall time that boys and girls spent in PA. While our report of no difference in overall PA volumes between girls and boys is congruent with other studies of PA in 2-year-olds [27], these results remain striking because studies also report differences in PA between girls and boys by 3 years of age [28]. Taken together, these findings encourage especial attention toward the influence of sociocultural and interpersonal factors on the differential trajectories of PA behavior in young girls and boys. To better understand the contextual qualifiers that may explain these apparent sex-based differences, further research on PA behaviors in young boys and girls is needed with a particular focus on the concurrent influences of dyadic interpersonal spatial dynamics, parental support for PA, and parenting style [29,30].

Regarding limitations of the study, the adult sample in our study was predominantly comprised of mothers and all the urban-dwelling families in our study reported an annual income below the federal poverty level, thus our results may be more indicative of activity–proximity relationships in mother-toddler dyads. Also, the use of dyadic analysis limits the direct comparison of these findings to other studies that have used a dyadic analysis approach to study child-parent PA behaviors [23]. Findings from our study may, therefore, be generalizable to other under-resourced toddler–parent dyads living in areas of pronounced need within a major urban center.

To our knowledge, this study is the first to use a dyadic analysis statistical methodology to analyze dyadic physical activity [4,9], as such parameter estimates were appropriately and necessarily adjusted for interdependent relationships between child and parent signals [23]. Along with the recent recommendation to include objective measures of dyadic proximity in studies of child-parent PA [9], we add that studies of dyadic PA and proximity should consider the use of dyadic analysis in the treatment of their dyadic data. Results from our study show its utility in analyzing correlated dyadic data, but also reveal its potential to uncover additional layers of information regarding cross-partner and within-subject PA behavior patterns. For example, an unintended, albeit notable, artifact of employing the hour-to-hour APIM was the discovery that toddlers’ hourly PA volumes were stable over time. Thus, while it is well-established that young children characteristically engage in random “short burst” PA patterns [12], our data show that toddlers’ activity patterns also appear to exhibit a significant degree of consistency throughout the day.

## 5. Conclusions

We found that child sex moderated the relationship between dyadic PA and proximity, and that children’s daily moderate–vigorous PA volumes were associated with characteristic differences in joint child-mother total PA. Girls engaged in more joint total PA time with their mothers than boys. Though child and mother time spent in moderate–vigorous PA were independent, children with ≥60 min of daily moderate–vigorous PA engaged in more daily joint child-mother PA time when compared to children with <60 min of daily moderate–vigorous PA. Daily moderate–vigorous PA volumes in toddlers were also associated with differences in relative distances between dyadic counterparts during joint PA. For boys in particular, participating in lower daily volumes of moderate–vigorous PA was associated with engaging in bouts of joint PA at relatively close distances to their mothers, as compared to more active boys and girls who engaged in joint PA at farther relative distances. These findings show that relative dyadic distance may be an important explanatory factor in characterizing dyadic PA. Results from our study invite further investigation of sex-based differences in the dyadic activity–proximity relationship across diverse samples of young children and parents.

## Figures and Tables

**Table 1:** Demographics, activity, inactivity, and spatial proximity in toddler–parent dyads attending an Early Head Start Program in a major metropolitan area.

	Dyads (*n* = 65)	
**Variables**	**Children**	**Parents**
Sex		
Female (*n*)	34	63
Male (*n*)	31	2
Age	29(4) months	32(6) years
Income below Federal Poverty Level (%)		100
Family Size (*n*)		4.1(1.2)
Country of Origin (%) (*n* = 58)		
Mexico		66.7%
Dominican Republic		6.8%
Ecuador		10.0%
USA		8.3%
Other		8.2%
Education (%) (*n* = 55)		
Less than High School		50.9%
High School Diploma/GED		29.1%
Associates Degree/Some College		10.9%
College Degree/Graduate Degree		9.1%
Accelerometer Wear time		
Days	5.5(1.5)	6.4(1.0)
Weekend days (*n* = 53)	1.4(0.8)	1.6(0.7)
Hours/day	10.1(1.3)	12.3(1.5)
Daily Minutes by Activity Intensity		
SED	312.6(61.3)	415.2(84.0)
TPA	293.7(60.1)	356.1(83.2)
MVPA	61.3(27.6)	27.2(15.9)
Meeting PA Guidelines (%)		
MVPA	44.6 ^a^	40.0 ^b^
TPA	98.5 ^c^	
Proximity Wear Time (*n* = 34)		
Days		6.4(1.1)
Hours/day		12.4(1.4)

Note: Table values are Mean (Std. Deviation) or Frequencies (%). Abbreviations: physical activity (PA), Sedentary Behavior (SED); Moderate–Vigorous Physical Activity (MVPA); Total Physical Activity (TPA). Activity Guidelines are: ^a^ American Heart Association, ^b^ American College of Sports Medicine, ^c^ Institute of Medicine.

**Table 2:** Mother-child activity and dyadic proximity characteristics stratified by child sex.

	Girl-Mother Dyads (*n* = 15)		Boy-Mother Dyads (*n* = 19)	
	Girls	Mothers	Boys	Mothers
Time (%) spent in each intensity while proximal				
SED	38(21)%	40(19)%	35(13)%	46(11)%
LPA	32(13)%	34(15)%	28(5)%	28(9)%
MVPA	5(4)%	3(3)%	7(4)%	3(3)%
Relative distance (z-scores)				
SED	0.07(0.17)	−0.13(0.33)	0.11(0.29)	−0.07(0.48)
LPA	0.23(0.16)	0.18(0.15)	0.12(0.29)	0.15(0.31)
MVPA	0.12(0.18)	0.24(0.19)	0.27(0.36)	−0.17(0.41)

Note: Table values are Median (IQR). All values are interpreted with respect to the indicated dyadic counterpart when their counterpart was proximally engaged in activity of any intensity. Percentages of time spent in proximity were calculated with respect to total wear time. Relative distances (z-scores) for dyadic counterparts: Nearer < 0; Farther > 0. Abbreviations: sedentary (SED), light physical activity (LPA), moderate–vigorous physical activity (MVPA).
